# Enhanced Antibacterial Activity of Vancomycin Loaded on Functionalized Polyketones

**DOI:** 10.3390/polym16131890

**Published:** 2024-07-02

**Authors:** Rachele Rampazzo, Andrea Vavasori, Lucio Ronchin, Pietro Riello, Martina Marchiori, Gloria Saorin, Valentina Beghetto

**Affiliations:** 1 Department of Molecular Sciences and Nanosystems, University Ca’ Foscari of Venice, Via Torino5 155, 30172 Venice, Italy; 2 Department of Architecture and Industrial Design, University of Campania “Luigi Vanvitelli”, 81031 Aversa, Italy; 3 Crossing S.r.l., Viale della Repubblica 193/b, 31100 Treviso, Italy; 4 Consorzio Interuniversitario per le Reattività Chimiche e la Catalisi (CIRCC), Via C. Ulpiani 27, 701268 Bari, Italy

**Keywords:** polyketone, Paal–Knorr, Vancomycin, antibiotics, drug delivery

## Abstract

Today, polymeric drug delivery systems (DDS) appear as an interesting solution against bacterial resistance, having great advantages such as low toxicity, biocompatibility, and biodegradability. In this work, two polyketones (PK) have been post-functionalized with sodium taurinate (PKT) or potassium sulfanilate (PKSK) and employed as carriers for Vancomycin against bacterial infections. Modified PKs were easily prepared by the Paal–Knorr reaction and loaded with Vancomycin at a variable pH. All polymers were characterized by FT-IR, DSC, TGA, SEM, and elemental analysis. Antimicrobial activity was tested against Gram-positive *Staphylococcus aureus* ATCC 25923 and correlated to the different pHs used for its loading (between 2.3 and 8.8). In particular, the minimum inhibitory concentrations achieved with PKT and PKSK loaded with Vancomycin were similar, at 0.23 μg/mL and 0.24 μg/mL, respectively, i.e., six times lower than that with Vancomycin alone. The use of post-functionalized aliphatic polyketones has thus been demonstrated to be a promising way to obtain very efficient polymeric DDS.

## 1. Introduction

Today, the fight against infectious diseases still poses a serious challenge worldwide for healthcare. According to the literature, bacteria are becoming quickly resistant to commercially available antibiotics and antiseptics [1,2,3,4,5,6,7]. Antibiotics are often used in massive doses to maintain their therapeutic effects because of their low bioavailability and unfavorable interactions with biological barriers, promoting bacterial resistance. Moreover, resistant bacteria develop different mechanisms, such as efflux pumps [8,9,10], the inactivation of enzyme expression [10,11,12], and the modification of drug penetration through barriers [10,13], further reducing the antibiotic efficiency. Additionally, bacteria may promote biofilm formation, which is a protective self-produced extracellular polymeric matrix, contributing to their difficult eradication [14,15,16]. Therefore, the in-depth comprehension of the resistance mechanisms and the development of alternative antimicrobials are necessary to address the problem of bacterial resistance [1,17,18,19,20,21,22].

Drug delivery systems (DDS) are widely used to prevent antibiotic and antiseptic resistance, improving drugs’ efficiency, selectivity, biodistribution, and pharmacokinetics and preventing biofilm formation [6,23,24,25,26,27,28,29,30,31,32]. Biocompatible materials, such as polysaccharides, proteins, lipids, and peptides, are widely used to produce DDS, avoiding drug degradation and adverse effects and controlling drug release [6,33,34,35,36,37,38].

Specifically devised polymers are also a promising alternative for biomedical and biotechnological applications, synthesized with different functional groups and promoting interactions with drugs [34,35,39,40,41,42,43,44,45,46]. DDS-based materials can be used as carriers of therapeutic molecules (nanoparticles, microparticles, implants) or as conjugates, bonding drug molecules through linkers designed to release them specifically at targeted sites [10,34,39,47,48,49,50,51]. To date, DDS prepared with biocompatible polymers have been widely described in the literature and many are also commercially available [52,53,54].

Within this area, aliphatic PK appear as an interesting yet rarely explored solution as polymeric carriers for DDS production [55,56]. In fact, aliphatic PK, produced by the catalytic copolymerization of CO and ethylene, may be easily post-functionalized by means of the so-called Paal–Knorr reaction [57], allowing one to obtain customized PK with specific desired functional groups [58,59,60,61,62,63,64,65,66,67,68,69,70,71]. Different examples have been reported of the direct condensation of 2-sulfanylethanol, hydroxylamine or furfural with ethylene and CO. Furthermore, in some cases, post-functionalized PK were shown to have high potential as supports for different types of enzymes [72], as germicide polymeric coatings [73], or as scaffolds for urothelial cells [56].

**Figure 1:**
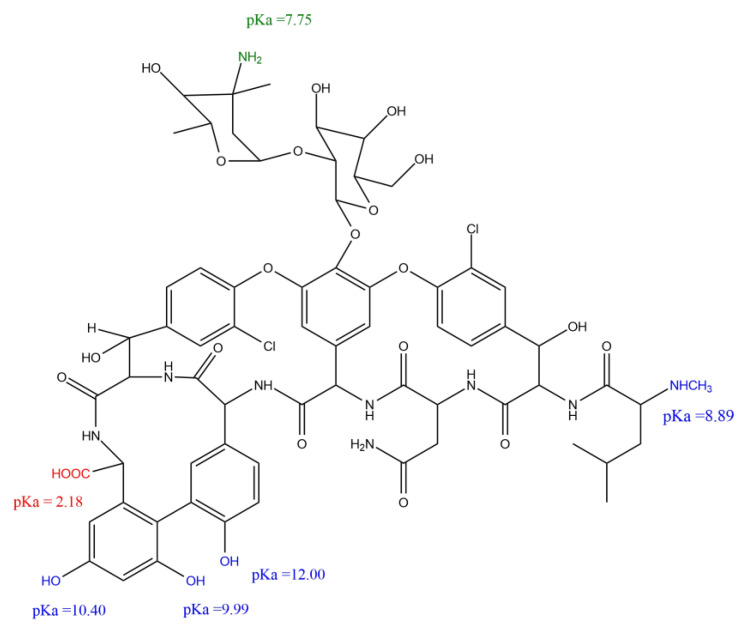
Molecular structure of Vancomycin [84].

Vancomycin (VCM), a branched tricyclic glycosylated peptide antibiotic (Figure 1), is a very interesting last-line-of-defense antibiotic used against serious infections caused by Staphylococci, Enterococci, and other Gram-positive pathogenic bacteria [74,75,76,77,78,79,80,81,82,83], inhibiting cell wall synthesis in sensitive bacteria. Because of its wide use, to limit the rise of bacterial resistance to VCM, the development of DDS is fundamental.

Physical–chemical studies have demonstrated that VCM encapsulated in polymeric PK formulations is able to penetrate the bacterial phospholipidic double membrane by releasing the drug locally, inhibiting biofilm formation and eradicating mature biofilms [14,38,85,86,87,88,89,90]. In this context, the aim of this work is to prepare polymeric DDS employing specifically customized PK able to interact with VCM through ionic and hydrophobic bonds [38,91,92]. PK were prepared according to a literature procedure [68], post-functionalized with potassium sulfanilate (PKSK) or sodium taurinate (PKT), and loaded with Vancomycin (VCM) at different pHs. All polymers were characterized by FT-IR, DSC, TGA, SEM, and elemental analysis, while NMR analysis was not performed due to the insolubility of PK, even after functionalization. The PKSK and PKT, before and after VCM loading, were further tested against the *Staphylococcus aureus* ATCC 25923 strain and their antimicrobial activity compared to that of pure VCM.

## 2. Materials and Methods

Carbon monoxide and ethene were supplied by the SIAD Company, Bergamo, Italy (“research grade”, purity > 99.9%). Bis(diphenylphosphino)propane (dppp), TsOH (*p*-toluene sulfonic acid), methanol (99.8%), diethyl ether (>99.5%), sulfanilic acid (≥98%), taurine (≥99%), sodium hydroxide (≥98%), potassium hydroxide (≥85%), *m*-cresol (99%), dimethyl sulfoxide (99%), agar, phosphate-buffered saline (PBS), nutrient broth (NB), Mueller–Hinton broth (MHB), and vancomycin hydrochloride were purchased from Sigma Aldrich Co. (St. Louis, MO, USA). MilliQ water was used for all experiments. The complexes [Pd(OAc)_2_(dppp)] were prepared as reported in the literature [93]. The *Staphylococcus aureus* ATCC 25923 strain was obtained from the American Type Culture Collection. The 96-well microtiter microplates were purchased from Thermo Fisher (Milan, Italy).

The average viscosity molecular weight of PK was evaluated as the limit viscosity number (LVN), using *m*-cresol as a solvent, and the viscosity was measured by a Cannon–Fenske-type capillary viscosimeter, thermostated at 25 °C, as reported in the literature [93].

FT-IR spectroscopy was performed on a Nicolet AVATAR Nexus FT-IR Thermo Fisher Scientific spectrometer in KBr, with 32 scans and a resolution of 4 cm^−1^ in the wave number range between 4000 cm^−1^ and 400 cm^−1^. The melting and decomposition temperature and thermogravimetric analysis of the polymers was performed using a DSC/TGA Linseis PTA ST1000 (Selb, Germany) under a nitrogen flow of 100 mL/min, with a temperature ramp of 20 °C min^−1^ from 30 °C to 500 °C. Elemental analyses for carbon, hydrogen, nitrogen, oxygen, and sulfur (CHNSO) were performed using a UNICUBE organic elemental analyzer (Elemental) (Tartu, Estonia). It offers highly sensitive analysis at a detection limit of 10 µg/g or 10 ppm. UV–Vis spectra were recorded on a UV Carey 100 (Agilent, Santa Clara, CA, USA) spectrophotometer in a glass cuvette in the range of 400–200 cm^−1^. Scanning electron microscopy (SEM) was carried out with a Zeiss Sigma|VP (Jena, Germany) variable-pressure instrument (VP-SEM), at 20 kV and variable magnifications. For SEM imaging, the samples were prepared by Au deposition (layer of about 40 nm) using AC sputtering.

Optical density measurement (OD600) was conducted using the BioTek Synergy H1, (Milano, Italy) a modular multimode microplate reader, with monochromator-based optics and filter-based optics. The optical density at 600 nm is referred to as OD600. The light at λ = 600 nm is easy to produce and does not damage or hinder microbial growth. 

### 2.1. Synthesis of Aliphatic Polyketones (PK)

The reaction was conducted in a 250 mL stainless-steel autoclave equipped with a 150 mL Pyrex glass beaker. In a typical experiment, 2.10 mg of [Pd(OAc)_2_(dppp)] (0.003 mmol), 246.23 mg of *p*-TsOH (1.290 mmol), and 60 mL of methanol were mixed in a 150 mL Pyrex glass beaker and then placed in the autoclave. The autoclave was then sealed and pressurized with a 1/1 mixture of CO/C_2_H_4_ at 5.0 MPa and heated to 90 °C for 4 h with stirring. After cooling to room temperature, the solid product was collected, filtered, and washed three times with methanol, acetone, and diethyl ether. The resulting white powder polyketone (PK) was dried in a vacuum for approximately 8 h [93,94,95,96]. M.p. 250 °C; FT-IR (KBr, cm^−1^): ν = 1690 (vs) (C=O), 2900 (s) (C-H), 1406 (s) (C-H), 1333 (s) (C-H), 1057 (s) (C-H). Elemental analysis (%) for PK found C 63.45, H 7.06, 0 29.49.

### 2.2. Synthesis of PKSK and PKT by Paal–Knorr Modification of PK with Potassium Sulfanilate or Sodium Taurinate 

In a round-bottom flask equipped with a mechanical stirrer and a reflux condenser, 1.00 g of polyketone (PK) was suspended in 30 mL of methanol with 1.88 g potassium sulfanilate (0.09 mol) or 1.3g sodium taurinate (0.09 mol). The reaction mixture was heated at 70 °C for 48 h and, after cooling, was collected by filtration and washed with methanol, acetone, and diethyl ether. The resulting brown powder polyketone with potassium sulfanilate (PKSK) and orange powder polyketone with sodium taurinate (PKT) were dried in a vacuum for 8 h and characterized by FT-IR, DSC, TGA, elemental analysis, SEM, and OD600 measurements [96,97,98,99,100].

PKSK: M.p. 234 °C; FT-IR data are reported in Table 1. Elemental analysis (%) for PKSK found C 51.18, H 5.38, N 4.02, S 9.31. 

PKT: M.p. 214 °C; FT-IR data are reported in Table 2. Elemental analysis (%) for PKT found C 54.76, H 6.5, N 1.66, S 4.23.

### 2.3. Loading of Vancomycin on PKSK or PKT

The loading of VCM on the functionalized polyketones (PKSK or PKT) following the same protocol and the sensitivity to pH were evaluated in DMSO solutions, in the 2.3–5.0–8.8 pH range (obtained by HCl or NaOH solutions, respectively) (pH 8+ DSA potentiometer, FAVS Scientific Equipment (Ferrara, Italy)) at room temperature. The polymer and Vancomycin, at a 2/1 wt/wt ratio, were suspended in 2mL DMSO (pH 2.3, 5.0, 8.8) under stirring. After 20 min, the solution was dialyzed against MilliQ water with Spectra/Por 1 Dialysis Membrane Standard RC Tubing MWCO at 6–8 kD for 24 h to remove the remaining unabsorbed antibiotic. The concentration of unabsorbed antibiotic (Free-VCM) found in the MilliQ water was measured using UV at a wavelength of 281 nm [101,102,103]. The VCM encapsulation efficiency % (EE%) and loading capacity % (LC%) were determined as follows:

$$EE\%=\frac{TotalVCM-FreeVCM}{TotalVCM}\times 100$$

$$LC\%=\frac{TotalVCM-FreeVCM}{Polymer+\left(TotalVCM-FreeVCM\right)}\times 100$$

Total VCM = the starting VCM amount used for the loading.

Polymer = the starting PKSK or PKT amount used for the loading considering no losses.

Samples were lyophilized and characterized by FT-IR, DSC, TGA, SEM, and OD600.

PKSK-VCM pH 2.3: M.p. 234 °C; FT-IR data are reported in Table 1. Elemental analysis (%) for PKSK-VCM pH 2.3 found C 50.87, H 4.96, N 4.36, S 7.67.

PKSK-VCM pH 5.0: M.p. 234 °C; FT-IR data are reported in Table 1. Elemental analysis (%) for PKSK-VCM pH 5.0 found C 47.38, H 3.87, N 7.13, S 2.37. 

PKSK-VCM pH 8.8: M.p. 234 °C; FT-IR data are reported in Table 1. Elemental analysis (%) for PKSK-VCM pH 8.8 found C 48.76, H 4.28, N 5.86, S 5.10.

PKT-VCM pH 2.3: M.p. 214 °C; FT-IR data are reported in Table 2. Elemental analysis (%) for PKT-VCM pH 2.3 found C 53.14, H 5.63, N 2.10, S 3.75.

PKT-VCM pH 5.0: M.p. 214 °C; FT-IR data are reported in Table 2. Elemental analysis (%) for PKT-VCM pH 5.0 found C 49.35, H 3.46, N 7.73, S 1.12.

PKT-VCM pH 8.8: M.p. 214 °C; FT-IR data are reported in Table 2. Elemental analysis (%) for PKT-VCM pH 8.8 found C 52.78, H 4.71, N 3.66, S 3.41.

### 2.4. Vancomycin Release

The Vancomycin release from PKT-VCM and PKSK-VCM was assessed using test tubes containing 5.0 mL of PBS pH 7.4 with 5.0 mg of the sample and thermostated at 37 °C. The experiments were carried out in triplicate (without stirring) under sink conditions. The released antibiotics were quantified by UV at a wavelength of 281 nm [84,99,103].

### 2.5. Antibacterial Activity

Antimicrobial activity was determined by the standard liquid dilution method in nutrient broth (NB) medium. *S. aureus* ATCC 25923 cells were grown overnight at 37 °C in NB broth and diluted in the same medium for assays. In a 96-well sterile microtiter tray, 50 µL of bacteria from overnight culture (adjusted to 1 × 10^6^ cells/mL) was added to the serial dilution of PKT-VCM and PKSK-VCM from 25 µg/mL and a final concentration of 0 mg/mL as a blank, in a total volume of 150 µL of NB. The 96-well microtiter tray was incubated at 37 °C, overnight, in a shaking incubator, and the cell growth was assessed by measuring the OD at 600 nm. Minimum inhibitory concentrations (MICs) were determined as the lowest amount of antibiotic.

## 3. Results and Discussion

### 3.1. Synthesis of Post-Functionalized PK

**Scheme 1:**
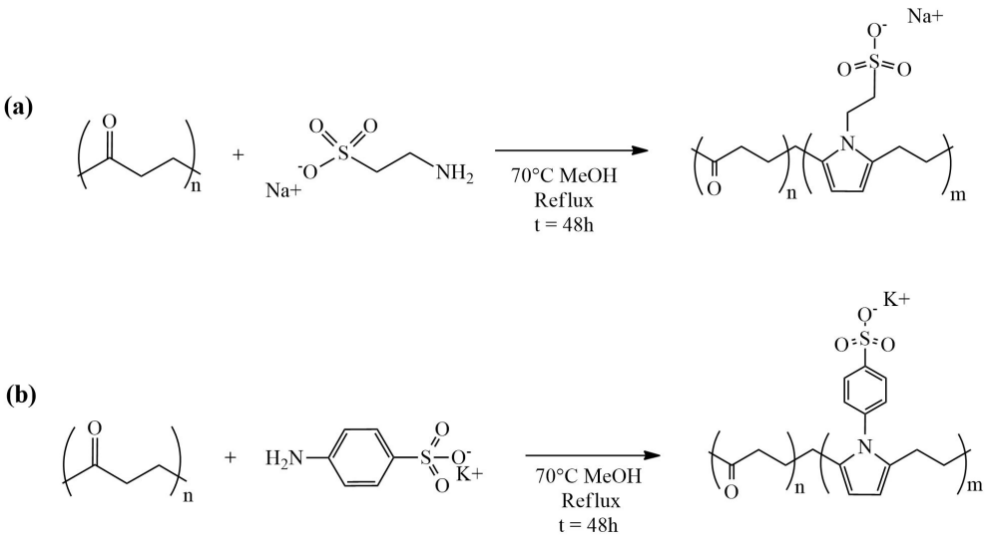
Synthetic scheme for the preparation of post-functionalized PK with (**a**) sodium taurinate (PKT) and (**b**) potassium sulfanilate (PKSK).

The PK prepared according to the literature method [68] (Mw = 15.272 g mol^−1^, determined as reported by Vavasori and coworkers [93]) was post-functionalized by the Paal–Knorr reaction in the presence of sodium taurinate or potassium sulfanilate (1/1 molar ratio) at 70 °C, for 48 h, in methanol as a solvent (Scheme 1a,b). After the work-up (see Section 2), the solid polymers, recovered in high yields (2.30 g, 80% and 1.96 g, 85%, respectively, for PKSK and PKT), were characterized by FT-IR, DSC, TGA, SEM, and elemental analysis. Sodium taurinate and potassium sulfanilate were chosen to prepare a specific modified PK (PKSK and PKT) containing anionic functional groups, which should promote the interaction and immobilization of VCM.

Moreover, it is interesting to note that sodium taurinate and potassium sulfanilate were expected to improve the solubility of the post-functionalized polymers, yet both PKSK and PKT were not soluble in any conventional organic solvent or in water (at any pH).

### 3.2. Influence of pH on Vancomycin Loading

When seeking the best loading conditions, it is important to consider that VCM is a large molecule bearing six different acidic and basic functional groups with specific pKa (Figure 1). Thus, VCM is differently charged, ranging from −4 at pH > 13 to +2 at an acidic pH (~0) [104]; consequently, its reactivity towards PKSK and PKT will be influenced by the pH.

The PKSK and PKT were loaded with VCM at room temperature using a common protocol and operating at three different pH values (2.3, 5.0, 8.8) [84]. The polymers loaded with VCM, named PKSK-VCM and PKT-VCM, were purified by dialysis and unabsorbed VCM measured by UV at 281 nm (Figure S1) [101,102,103], to determine the encapsulation efficiency (EE%) and loading capacity (LC%) (reported in Table 3; all experiments were performed in triplicate and all data are expressed as the mean ± standard deviation).

The data reported in Table 3 show that, with both polymers, the maximum LC% and highest EE% were achieved at pH 5.0. These data were further confirmed by the elemental analysis, since the highest nitrogen content was determined at pH 5.0, confirming the presence of higher amounts of Vancomycin. At pH 2.3 or 8.8, a decrease in EE% of up to 34% for PKSK-VCM and 57% for PKT-VCM and in LC% between 8% and 21%, respectively, was registered. According to the literature, at pH 2.3, VCM has a higher positive charge (+2); therefore, this pH was supposed to be the optimal one for the loading on the anionic polyketones tested [104]. Nonetheless, the data reported in Table 3 clearly show that the highest VCM loading was obtained at pH 5.0, confirming that not only ionic but also hydrophobic interactions are important to promote VCM loading. Moreover, the higher encapsulation efficiency of PKSK-VCM as compared to PKT-VCM may be attributed to the stronger hydrophobic interactions between VCM and the aromatic functional groups present in PKSK [38,91,92,105].

### 3.3. PKT-VCM and PKSK-VCM Characterisation

PKT, PKSK, PKT-VCM, and PKSK-VCM were lyophilized and characterized by FT-IR, DSC, TGA, SEM, and elemental analysis. The FT-IR of PKSK, PKT, PKT-VCM, and PKSK-VCM, prepared at different pHs, showed modest differences, and no clear evidence of the presence of VCM in the FT-IR of PKSK-VCM and PKT-VCM could be highlighted, probably due to the very low amount of antibiotic present with respect to the polymer. Nevertheless, the comparison of the FT-IR of the polymers before and after VCM loading shows a slight shift in the peaks in the range of 1010–1095 cm^−1^, corresponding to the SO_3_^−^ groups, which are the functional groups most affected by the loading of VCM (see Figure S2 and Table 1 and Table 2). Additionally, the peaks corresponding to the aromatic rings present in PKSK also show a modest shift (813–832 cm^−1^ to 806 cm^−1^) (see Figure S2 and Table 1 and Table 2), suggesting that not only ionic but also hydrophobic interactions may contribute to drug retention [38,91].

To gain further information on the characteristics of the polymers prepared, thermal analyses (DSC, TGA) were performed (Figures S3 and S4). In agreement with the literature [106], due to the inferior amount of VCM used as compared to the polymer, no significant difference was observed between PKT and PKT-VCM or PKSK and PKSK-VCM.

**Figure 2:**
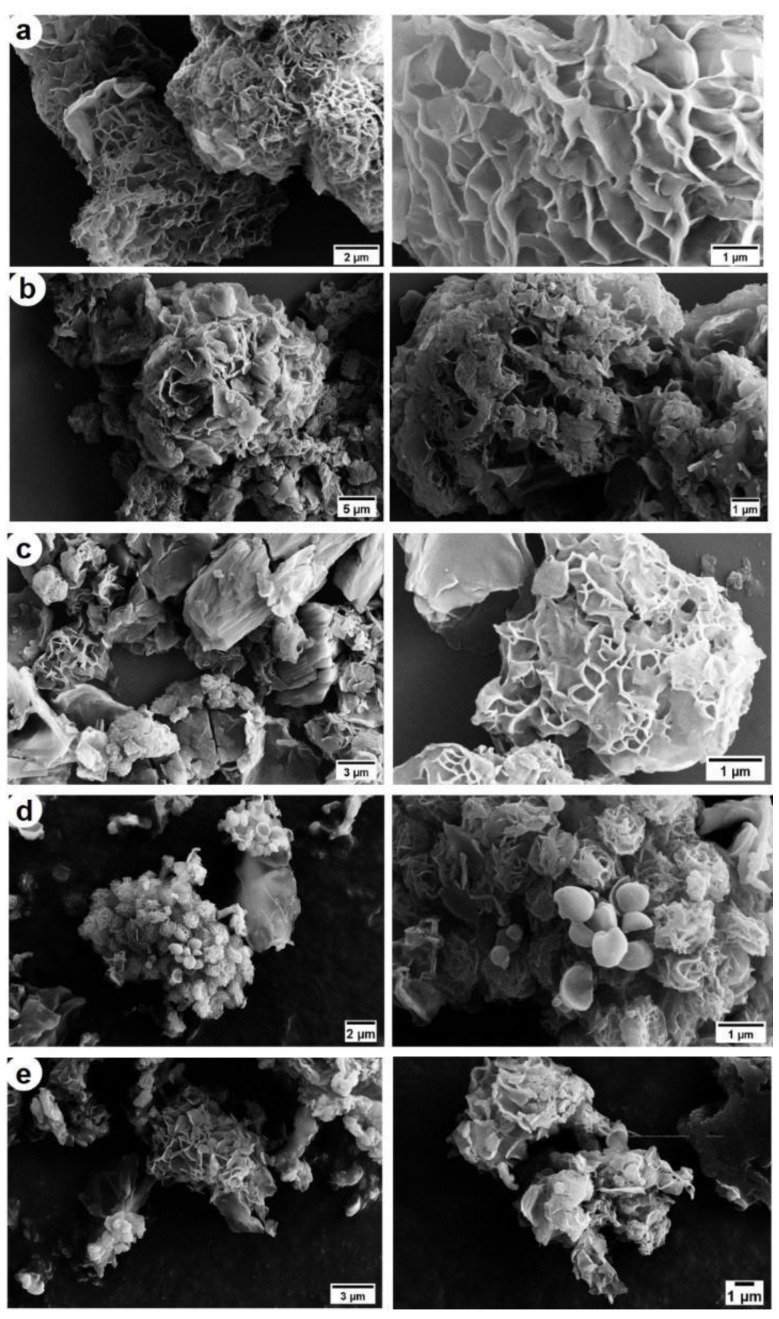
SEM images of (**a**) PK, (**b**) PKT, (**c**) PKSK, (**d**) PKT-VCM (pH 5.0), (**e**) PKSK-VCM (pH 5.0).

Interestingly, the SEM analysis of PKT-VCM and PKSK-VCM with higher VCM loading (Table 3) showed evident differences between the polymers before and after VCM loading (Figure 2). The SEM images of the PK (Figure 2a) show pores with lamellate edges with an average size of 1.13 ± 0.05 μm, while, on the PKT surface (Figure 2b), the pores are smoother and less laminated, with poorly defined edges (0.48 ± 0.03 μm), probably due to the functionalization of the PK with sodium taurinate, leading to morphological changes. Similarly, the PKSK images (Figure 2c) showed a different morphology compared to the PK, due to the partial functionalization, although, in this case, pores were also visible (0.25 ± 0.02 μm). When VCM was loaded on the PKT or PKSK (Figure 2d and Figure 2e, respectively), the morphology of the polymers further changed. New particles with a smoother and rounder surface, with spherical geometry for PKT-VCM, were observed on the polymeric surface (PKT-VCM 0.64 ± 0.04 μm; PKSK-VCM 0.43 ± 0.04 μm). These particles were probably due to the presence of VCM in the samples.

### 3.4. Release of Vancomycin from PK Polymers

**Figure 3:**
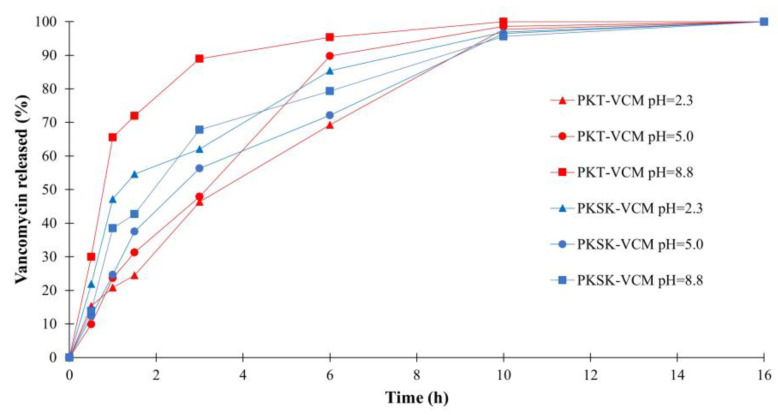
Vancomycin release at pH 7.4 from PKSK-VCM and PKT-VCM prepared at different pHs (2.3, 5.0, 8.8).

Different polymeric samples of PKSK and PKT loaded with VCM at different pHs (PKT-VCM 2.3, 5.0, 8.8 and PKSK-VCM 2.3, 5.0, 8.8; see Figure 3) were employed for the study of VCM release at physiological pH 7.4, analogously to the procedure reported by Ruiz et al. for a formulation of VCM on surface-modified polypropylene [84]. According to the data reported in Figure 3, it emerges that PKSK-VCM’s release trends are rather similar, while a wider gap exists for the release trends of PKT-VCM. Further, the release of VCM from PKSK-VCM 5.0 is slower compared to that of PKSK-VCM 2.3 and 8.8, further suggesting the higher strength of the interactions formed between PKSK and VCM at this specific pH. In contrast, the release trends of VCM from PKT-VCM prepared at different pHs show larger differences compared to those of PKSK-VCM, and the slowest release was obtained with PKT-VCM prepared at pH 2.3.

The difference in the VCM release profiles between the PKSK and PKT could be ascribed to the different chemical compositions of the post-functionalized polyketones. In fact, the PKSK and PKT were deliberately modified with different functional groups (all bearing an SO^3−^) but with different chemical backbones to highlight possible differences between the aromatic (PKSK) and aliphatic (PKT) functionalities present. Some minor differences between the two polymers were evidenced by FT-IR, but the release tests clearly highlighted the importance of the functional group present on the polymer. Thus, not only ionic but also hydrophobic interactions between the aromatic functionalities of sulfanilate and VCM contribute to strengthening the interaction between the drug and the polymer.

### 3.5. Antimicrobial Tests

To test PKSK-VCM and PKT-VCM’s antibacterial activity, minimal inhibitory concentration (MIC) analyses were performed against Gram-positive bacterial strain *Staphylococcus aureus* ATCC 25923 (MRSA), one of the main pathogens that can form biofilm infections [107,108]. Preliminarily tests against *Staphylococcus aureus* with PK, PKSK, and PKT, at concentrations between 0.2 and 2.0 log (µg/mL), showed the total inefficiency of the polymers against this bacterium (Figure S5).

The data obtained from the antimicrobial tests carried out in the presence of PKSK-VCM and PKT-VCM are reported in Table 4 (with corresponding growth curves in Figure S6). These data once more clearly show the significant dependence of the efficiency of VCM loading on the pH. In particular, loading at pH 5.0 allowed us to achieve MIC values ~six times lower than that of VCM alone, with both polymers, while PKSK-VCM and PKT-VCM loaded at pH 2.3 and 8.8 showed MIC values comparable to or higher than that of VCM alone.

## 4. Conclusions

In this work, VCM has been successfully loaded on two polymers (PKT, PKSK) obtained from PK modified with the Paal–Knorr reaction using sodium taurinate (T) and potassium sulfanilate (SK). The loading of VCM was studied at three different pHs, showing a substantial influence on the characteristics of the final polymeric formulation. Indeed, for both polymers, the best EE% and LC% were obtained at pH 5.0 (for PKSK EE% 75.00, LC% 31.03; PKT EE% 80.00, LC% 31.58), together with prolonged release. Interestingly, the MIC values obtained with PKSK-VCM and PKT-VCM were six times lower than those of VCM alone. These optimal achievements might be the result of different interactions between the VCM and polymers, which were supposed to involve both ionic and hydrophobic interactions. Moreover, the pH used for loading has proven to be a very important variable, probably affecting the interactions occurring between the drug and the polymer. These results confirm the promising role of functionalized polyketones as DDS for VCM delivery; therefore, further studies are ongoing to understand the influence of the polymer structure in order to enhance the solubility and the loading mechanism. Moreover, the loading of different antibiotics on these polymers and their antibacterial activity will also be investigated.

## Figures and Tables

**Table 1:** FT-IR data of PKSK and PKSK-VCM samples at pH 2.3, 5, 8.8.

PKSK	PKSK-VCM pH 2.3	PKSK-VCM pH 5	PKSK-VCM pH 8.8	Functional Groups	Note
Wavenumbers (cm^−1^)					
685	x	x	x	ẟ C=C ẟ C=C-H	Strong
813, 832	806	806	806	ẟ C=C-H ẟ C=C	Weak
1010, 1035, 1057	1015, 1035, 1057	1015, 1035, 1057	1015, 1035, 1057	ν S=O ν C-N ẟ C=C-H	Change in relative intensity
1124	1124	1124	1124	ν C-N	
1164	~1195	~1195	~1195	ν C-N ν C=O	Sharp for PKSK, broad for all other samples
1246, 1259	1260	1260	1260	ν C=O ν C-N	Change in relative intensity
1335	1335	1335	1335	ν S=O ẟ C-H	
x	x	1384	1384	ν S=O	
1408, 1426	1408, 1426	1408, 1426	1408, 1426	ν S=O ν C=C-H	
1499	1499	1499	1499	ẟ C=C-H	Change in relative intensity
1693	1693	1693	1693	ν C=O	
2852, 2912, 2963	2852, 2912, 2963	2852, 2912, 2963	2852, 2912, 2963	ν C-H	
3437	3437	3437	3437	ν OH	

**Table 2:** FT-IR data of PKT and PKT-VCM samples at pH 2.3, 5, 8.8.

PKT	PKT-VCM pH 2.3	PKT-VCM pH 5	PKT-VCM pH 8.8	Functional Groups	Note
Wavenumbers (cm^−1^)					
812	812	812	812	ẟ C=C-H ẟ C=C	
1054	1023, 1054, 1095	1023, 1054, 1095	1023, 1054, 1095	ν S=O ν C-N ẟ C=C-H	1023 and 1095 shoulders of the central peak
1198	~1198	~1198	~1198	ν C-N ν C=O	Broader for PKT-VCM samples
1259	1259	1259	1259	ν C=O ν C-N	
1336	1336	1336	1336	ν S=O ẟ C-H	
x	1384	1384	1384	ν S=O	
1409, 1427	1409, 1427	1409, 1427	1409, 1427	ν S=O ν C=C-H	
1470	1470	1470	1470	ẟ C=C-H	Weak and broad change in relative intensity
1693	1693	1693	1693	ν C=O	
2853, 2913, 2953	2852, 2918, 2962	2852, 2918, 2962	2852, 2918, 2962	ν C-H	
~3439	~3439	~3439	~3439	ν OH	

**Table 3:** Elemental analysis of PKT, PKSK, PKT-VCM, and PKSK-VCM. EE% and LC% of PKSK-VCM and PKT-VCM.

Sample	pH	Elementals (%)		EE%	LC%
		N	S		
PKSK	-	4.02 ± 0.01	9.31 ± 0.02	-	-
PKSK-VCM	2.3	4.36 ± 0.02	7.67 ± 0.01	41.25 ± 0.03	23.08 ± 0.01
PKSK-VCM	5.0	7.13 ± 0.01	2.37 ± 0.03	75.00 ± 0.02	31.03 ± 0.01
PKSK-VCM	8.8	5.86 ± 0.01	5.10 ± 0.01	66.66 ± 0.01	25.00 ± 0.02
PKT	-	1.66 ± 0.03	4.23 ± 0.02	-	-
PKT-VCM	2.3	2.10 ± 0.01	3.75 ± 0.03	23.08 ± 0.01	10.71 ± 0.02
PKT-VCM	5.0	7.73 ± 0.02	1.12 ± 0.01	80.00 ± 0.02	31.58 ± 0.01
PKT-VCM	8.8	3.66 ± 0.03	3.41 ± 0.03	42.67 ± 0.02	15.15 ± 0.03

**Table 4:** MIC values for PKSK-VCM and PKT-VCM loaded at variable pH, tested against *Staphylococcus aureus* ATCC 25923 (MRSA).

Sample	pH	MIC (µg/mL)
PKSK-VCM	2.3	1.44
PKSK-VCM	5.0	0.23
PKSK-VCM	8.8	1.56
PKT-VCM	2.3	2.68
PKT-VCM	5.0	0.24
PKT-VCM	8.8	3.79
VCM		1.54

## Data Availability

The original contributions presented in the study are included in the article/Supplementary Materials; further inquiries can be directed to the corresponding authors.

## Supplementary Materials

The following supporting information can be downloaded at: https://www.mdpi.com/article/10.3390/polym16131890/s1, Figure S1: UV-Vis spectra obtained for the calibration plot together with two example of samples (PKSK-VCM pH5.0 and PKSK-VCM pH 5.0) used for the quantification; Figure S2: FT-IR spectra of VCM and (A) PKT, PKT-VCM at pH 2.3, 5.0, 8.8 samples and (B) PKSK, PKSK-VCM at pH 2.3, 5.0, 8.8 samples; Figure S3: DSC of VCM and (A) PKSK, PKSK-VCM at pH 2.3, 5.0, 8.8 samples and (B) PKT, PKT-VCM at pH 2.3, 5.0, 8.8 samples; Figure S4: TGA of VCM and (A) PKSK, PKSK-VCM at pH 2.3, 5.0, 8.8 samples and (B) PKT, PKT-VCM at pH 2.3, 5.0, 8.8 samples; Figure S5: Growth curves of bacteria (expressed as normalized values of OD600) in presence of PK, PKT, PKSK at various concentrations (µg/mL); Figure S6: Growth curves of bacteria (expressed as normalized values of OD600) in presence of VCM at various concentrations (µg/mL) for PKSK (above) and PKT (below) samples at the different loading pH (2.3, 5.0, 8.8).
