# Supplementary material for: Enhanced Antibacterial Activity of Vancomycin Loaded on Functionalized Polyketones
Source: Polymers (Basel). 2024 Jul 2;16(13):1890. doi: 10.3390/polym16131890 (PMC11244503; doi:10.3390/polym16131890)
Supplement: Supplementary file 1 [file polymers-16-01890-s001.zip › polymers-3086653-supplementary.pdf]

Enhanced Antibacterial Activity of Vancomycin Loaded on Functionalized Polyketones

Rachele Rampazzo [1,2], Andrea Vavasori [1,*], Lucio Ronchin [1], Pietro Riello [1], Martina Marchiori [1], Gloria Saorin [1] and Valentina Beghetto [1,3,4,*]

[1] Department of Molecular Sciences and Nanosystems, University Ca' Foscari of Venice, Via Torino5 155, 30172 Venice, Italy; rachele.rampazzo@unive.it (R.R.); ronchin@unive.it (L.R.); riellop@unive.it (P.R.); marmarti@unive.it (M.M.); gloria.saorin@unive.it (G.S.)
[2] Department of Architecture and Industrial Design, University of Campania "Luigi Vanvitelli", 81031 Aversa, Italy
[3] Crossing S.r.l., Viale della Repubblica 193/b, 31100 Treviso, Italy
[4] Consorzio Interuniversitario per le Reattività Chimiche e la Catalisi (CIRCC), Via C. Ulpiani 27, 701268 Bari, Italy
* Correspondence: andrea.vavasori@unive.it (A.V.); beghetto@unive.it or valentina.beghetto@crossing-srl.com (V.B.); Tel.: +39−0412348928 (V.B.)

SUPPORTING INFORMATIONS

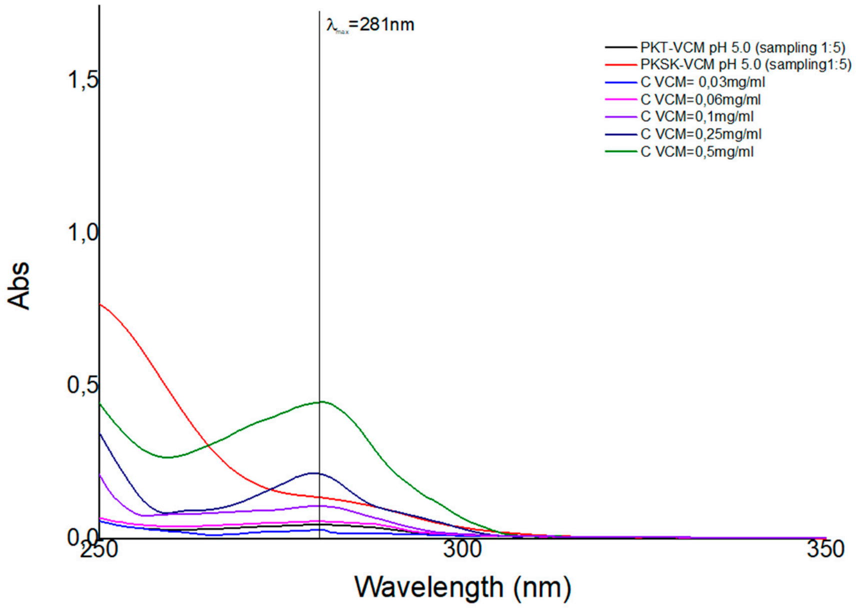

Figure S1. UV-Vis spectra obtained for the calibration plot together with two example of samples (PKSK-VCM pH5.0 and PKSK-VCM pH 5.0) used for the quantification.

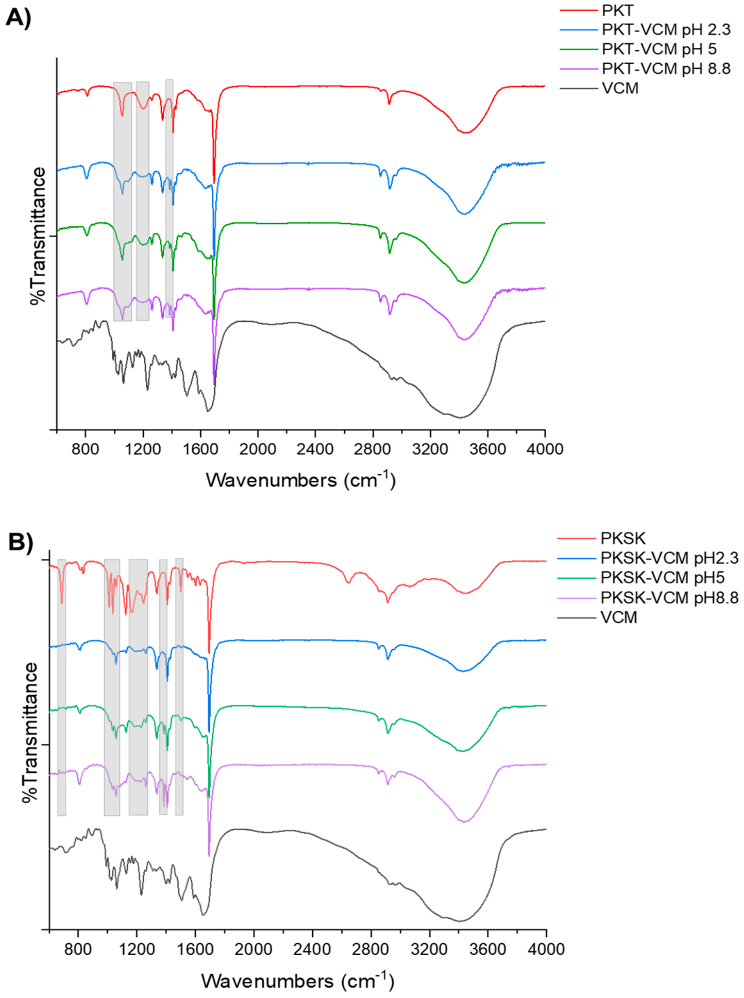

Figure S2. FT-IR spectra of VCM and A) PKT, PKT-VCM at pH 2.3, 5.0, 8.8 samples and B) PKSK, PKSK-VCM at pH 2.3, 5.0, 8.8 samples.

A)

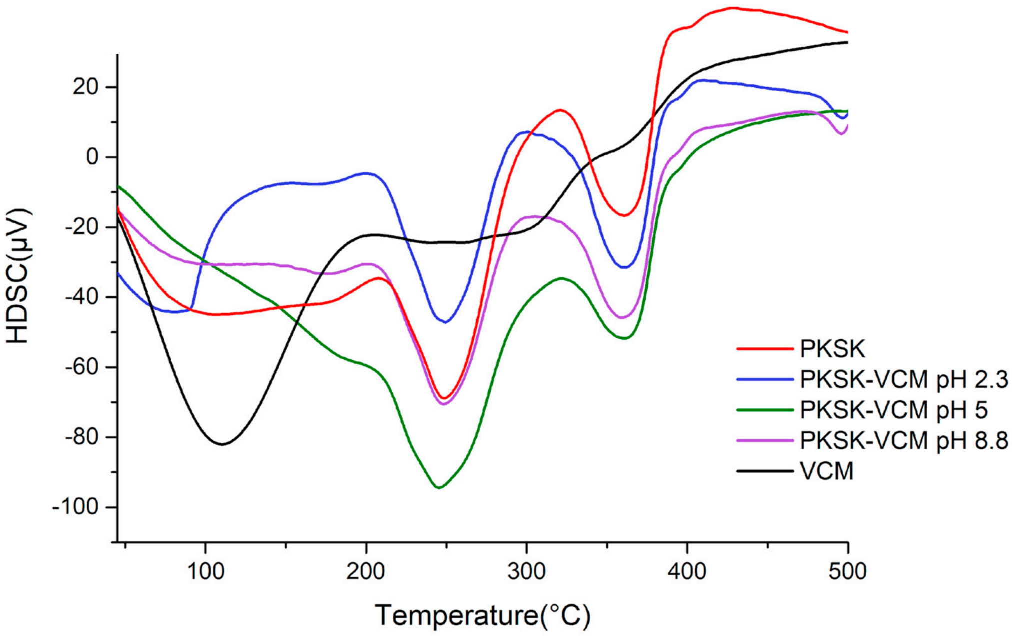

B)

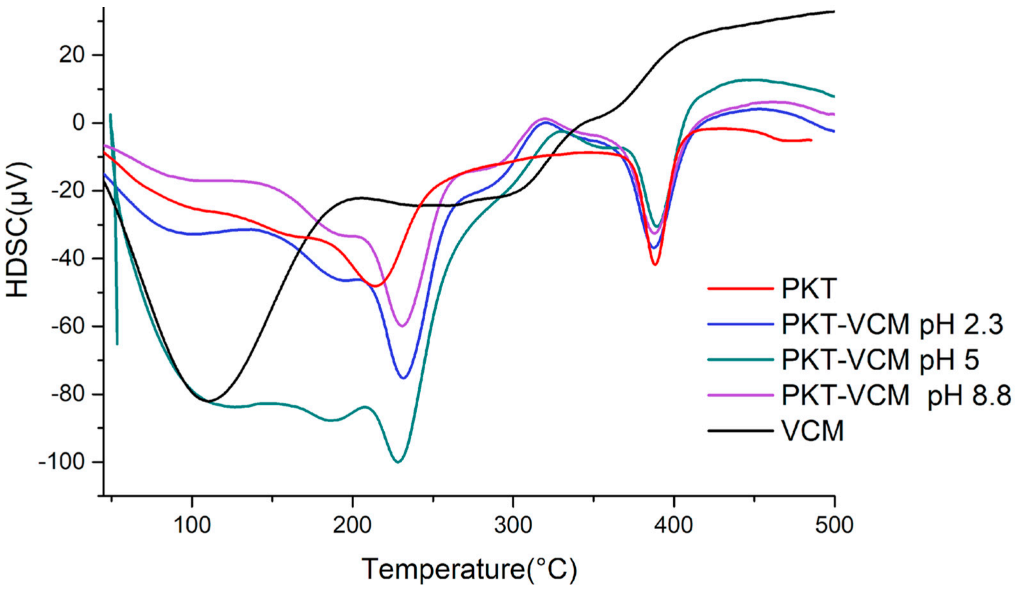

Figure S3. DSC of VCM and A) PKSK, PKSK-VCM at pH 2.3, 5.0, 8.8 samples and B) PKT, PKT-VCM at pH 2.3, 5.0, 8.8 samples.

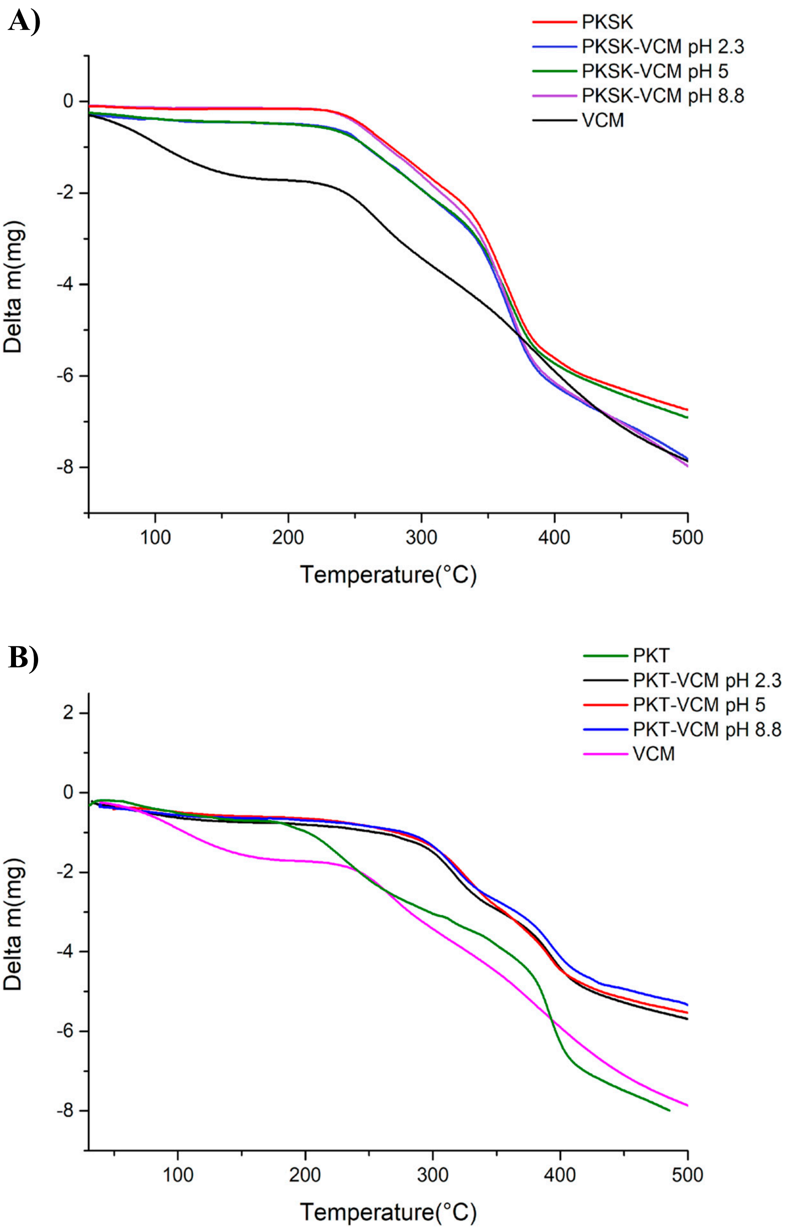

Figure S4. TGA of VCM and A) PKSK, PKSK-VCM at pH 2.3, 5.0, 8.8 samples and B) PKT, PKT-VCM at pH 2.3, 5.0, 8.8 samples.

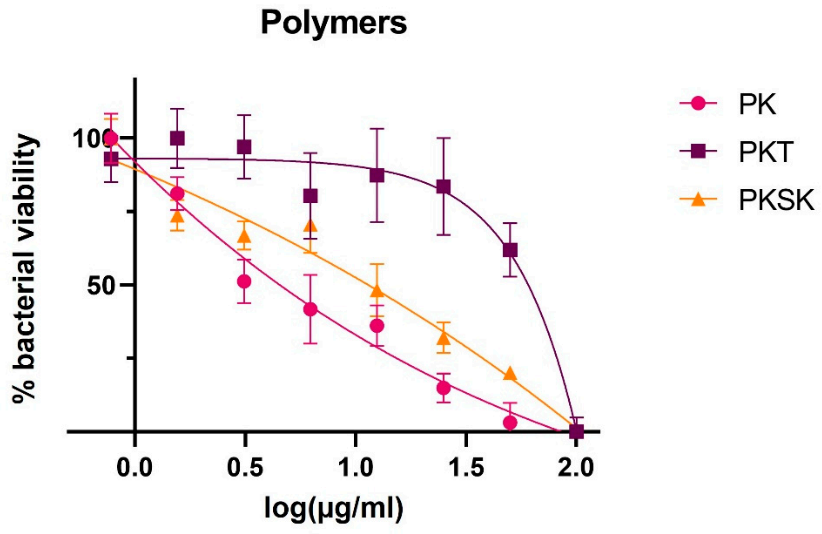

Figure S5. Growth curves of bacteria (expressed as normalized values of OD600) in presence of PK, PKT, PKSK at various concentrations (μg/mL).

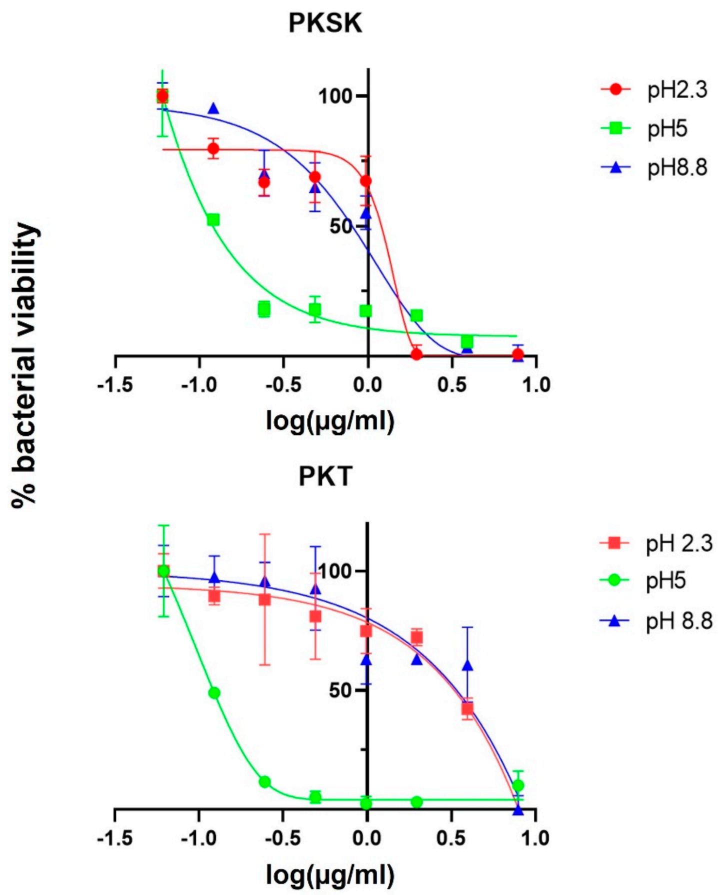

Figure S6. Growth curves of bacteria (expressed as normalized values of OD600) in presence of VCM at various concentrations (μg/mL) for PKSK (above) and PKT (below) samples at the different loading pH (2.3, 5.0, 8.8).
